# Phenotypic Expression of Respiratory Diseases and Tailored Treatment in Patients with Intermediate Alpha-1 Antitrypsin Deficiency: Evidence from a Retrospective Analysis of a Selected Cohort of Patients

**DOI:** 10.3390/medicina61101747

**Published:** 2025-09-25

**Authors:** Anna Annunziata, Giuseppe Fiorentino, Francesca Simioli, Lidia Atripaldi, Marco Balestrino, Giacomo Zuccarini, Barbara Piras, Alessandro Libra, Fabio Pino, Pierpaolo Di Micco, Carmine Siniscalchi, Ilaria Ferrarotti, Luigi Aronne, Raffaella Manzo, Carlo Vancheri, Cecilia Calabrese

**Affiliations:** 1 Respiratory Pathophysiology and Rehabilitation Unit, Azienda Ospedaliera di Rilievo Nazionale dei Colli, Monaldi Hospital, 80131 Naples, Italy; anna.annunziata@gmail.com (A.A.); giuseppe.fiorentino@ospedalideicolli.it (G.F.); francesimioli@gmail.com (F.S.); lidiett91@gmail.com (L.A.); giggiaronne@gmail.com (L.A.); raffaella.manzo@ospedaledeicolli.it (R.M.); 2 Department of Translational Medical Sciences, University of Campania “Luigi Vanvitelli”, Azienda Ospedaliera di Rilievo Nazionale dei Colli, Monaldi Hospital, 80131 Naples, Italy; marcobalestrino.mb@gmail.com (M.B.); cecilia.calabrese@unicampania.it (C.C.); 3 Respiratory Diseases Unit, Ospedale Santo Spirito, 65124 Pescara, Italy; gzuccarini_83@yahoo.it; 4 Respiratory Unit, Department of Medical, Surgical and Experimental Sciences, University of Sassari, Via San Pietro, 07100 Sassari, Italy; barbara.piras@aouss.it; 5 Department of Clinical and Experimental Medicine, Policlinico “G. Rodolico-San Marco”, University of Catania, 95123 Catania, Italy; alessandrolibra@outlook.it (A.L.); pinofabio@outlook.it (F.P.); carlo.vancheri@unict.it (C.V.); 6 UOC Medicina Interna, P.O. Santa Maria delle Grazie, Pozzuoli, ASL Napoli2 Nord, 80076 Naples, Italy; pdimicco@libero.it; 7 Internal Medicine Department, Parma University Hospital, 43126 Parma, Italy; 8 Department of Internal Medicine and Therapeutics, Pulmonology Unit, University of Pavia, 27100 Pavia, Italy; ilaria.ferrarotti@unipv.it

**Keywords:** alpha-1 antitrypsin deficiency, intermediate deficiency, rare AATD variants, augmentation therapy, quality of life, pulmonary exacerbation

## Abstract

*Introduction*: Alpha-1 antitrypsin deficiency (AATD) is a genetic condition caused by SERPINA1 variants with variable severity. Current international guidelines do not recommend augmentation therapy for intermediate AATD; nevertheless, some patients show clinically severe phenotypes in real-world practice. We aimed to evaluate, in an exploratory manner, the potential effects of augmentation therapy on exacerbations, quality of life, and lung function in this subgroup. *Methods*: In this multicenter retrospective study, we included 27 heterozygous patients with intermediate AATD (serum AAT 50–110 mg/dL), Chronic Obstructive Pulmonary Disease (COPD), and/or emphysema. Clinical phenotypes included emphysema-predominant disease, COPD with frequent exacerbations, and overlap with bronchiectasis/asthma; HRCT patterns were recorded. We assessed the annual number of exacerbations (moderate: steroids/antibiotics; severe: hospitalization/including pneumothorax), St. George’s Respiratory Questionnaire (SGRQ), and lung function before and after 12 months of therapy. *Results*: Augmentation therapy was associated with a reduction in annual exacerbations from a median (IQR) of 2 (1.5–3) to 1 (0–1) (*p* < 0.0001) and an improvement in SGRQ total score (58.89 ± 16.83 to 48.34 ± 21.20; *p* = 0.0039). The mean SGRQ change exceeded the 4-point MCID for COPD. No significant changes were observed in spirometry or Diffusing Capacity of the Lung for Carbon Monoxide (DLCO). *Conclusions*: These exploratory findings suggest that augmentation therapy may reduce exacerbations and improve quality of life in selected patients with intermediate AATD and COPD/emphysema. Given the retrospective design, small sample, and lack of a control group, the results should be interpreted as hypothesis-generating and warrant confirmation in prospective studies.

## 1. Introduction

Alpha-1 antitrypsin (AAT) deficiency (AATD) is an autosomal codominant disorder due to SERPINA1 variants leading to reduced or dysfunctional AAT and increased protease activity in the lung [1,2,3]. The PI*M allele is the normal variant, whereas S and Z are the most frequent deficient variants; null alleles abolish protein production and confer the highest risk for severe disease [4,5,6]. Historically, several rare variants have been named after the sites where they were first identified, but their quantitative/functional impact is heterogeneous [3,5]. AAT inhibits neutrophil elastase and other proteases, limiting connective-tissue damage and modulating inflammation and immunity [7,8,9,10,11].

Intermediate AATD is commonly defined by serum AAT levels of 50–110 mg/dL, typically in simple heterozygotes [7,8]. Despite “intermediate” biochemistry, some patients—especially smokers or ex-smokers—develop clinically significant COPD/emphysema and frequent exacerbations [12,13,14,15,16]. Current guidelines generally restrict augmentation therapy to severe AATD (AAT ≤ 50 mg/dL) with functional impairment or accelerated decline, and do not recommend treatment for intermediate deficiency [17,18,19,20,21,22]. This leaves a clinical gap for real-world patients with intermediate AATD but severe phenotypes.

The only specific treatment for emphysema associated with alpha-1 antitrypsin deficiency (AATD) is intravenous infusion of purified AAT from pooled human plasma. This product was developed in the United States and approved by the U.S. Food and Drug Administration in 1987. In Italy there are only two drugs available to perform replacement therapy in patients with different AATD deficiency: Prolastin^®^ (Grifols Deutchsland GmbH, Germany) and Respreeza^®^ (CSL Behring GmbH, Germany). Both are indicated for augmentation treatment in patients with severe AATD (PiZZ, PiZ null, Pi null, and PiSZ genotypes) and pulmonary emphysema. The recommended dose is 60 mg/kg/week administered at an infusion rate of 0.08 mL/kg/min.

In the present paper, we collected clinical cases from different Italian reference AATD centers, in which medical doctors prescribed replacement therapy with Prolastin^®^ and Respreeza^®^ to COPD patients with pulmonary emphysema and clinical overt exacerbation affected by intermediate AATD, caused by a single pathogenic allele. The primary endpoint of the study was to evaluate the clinical efficacy of 12 months of augmentation therapy on the exacerbation rates of the disease. Secondary endpoints were the effects on quality of life and respiratory function.

## 2. Materials and Methods

We retrospectively evaluated clinical and functional data of patients affected by AATD-related lung diseases with intermediate serum levels of AAT and a simple heterozygous genotype, who received replacement therapy due to their clinical/functional or radiological conditions, during follow-ups at various Italian centers:

- Unit of Pathophysiology and Respiratory Rehabilitation Monaldi Hospital, Naples;
- Regional Reference Centre for the Prevention, Diagnosis and Treatment of Rare Lung Diseases, University of Catania;
- Unit of Pneumology, Hospital of Pescara;
- Unit of Clinical and Interventional Pulmonology, Hospital of Sassari.

Diagnosis followed the previously published diagnostic algorithm for AATD, including serum AAT by nephelometry (CRP-adjusted), Pi-typing/genotyping, and confirmatory testing [23], and all gave their written informed consent to be involved in this study.

Given the rarity of intermediate AATD cases eligible for augmentation therapy, a multicenter retrospective design was chosen to maximize sample size and capture real-world treatment patterns. This was a multicenter retrospective cohort with consecutive sampling across the four Italian centers. Patients were identified from local AATD clinics, infusion registries, and electronic medical records, and screened against prespecified eligibility criteria.

Patients were included whether they were on optimized therapy for at least 12 months before the start of the replacement/augmentation therapy and a complete clinical functional and radiological assessment (medical history and physical examination; exacerbation and smoking history with pack-years; spirometry [FEV1, FVC, FEV1/FVC]; single-breath DLCO; and HRCT reporting emphysema distribution/airway involvement) was available. Active smokers and patients with malignancies or unstable heart, kidney, and liver diseases were excluded from the study.

Rationale: ongoing smoke exposure is a major phenotype modifier in AATD; therefore, excluding current smokers minimized confounding. Ex-smokers and never-smokers were eligible, and pack-years were recorded.

All patients underwent replacement therapy at a dosage of 60 mg/kg every week.

Commercial products used were Prolastin^®^ and Respreeza^®^, administered per label at 60 mg/kg weekly; outcomes were pooled irrespective of product, as dose and indications are equivalent, and product-specific comparisons were beyond the study aims.

At Time 0 (first evaluation), the following data were collected:

- Demographic characteristics (age and gender);
- Smoking habit (ex-smokers and never smokers; pack-years);
- Serum AAT levels, quantified by nephelometry.

Endpoints: The primary endpoint was the change in annual exacerbation frequency (moderate = systemic corticosteroids/antibiotics; severe = hospitalization, with pneumothorax a priori classified as severe). Secondary endpoints were changes in SGRQ (total and domains; MCID 4 points) and lung function (FEV1, FEV1/FVC, DLCO).

At T1 (replacement therapy beginning) and T2 (12 months after beginning AAT therapy), we evaluated:

- The number of exacerbations in the previous year: Exacerbations were defined as an acute worsening of respiratory symptoms (sputum volume, sputum purulence, and breathlessness) that resulted in additional therapy with systemic corticosteroids and antibiotics (moderate) or hospitalizations (severe), according to GOLD recommendations [24]. Pneumothorax episodes were a priori classified as severe events because they invariably led to acute respiratory deterioration requiring hospital-based interventions, thus meeting our ‘severe’ criterion (hospitalization). Quality of life measured with St. George’s Respiratory Questionnaire (SGRQ), an instrument designed to measure the health impairment in patients with obstructive airway diseases. Four scores were calculated: (1) symptoms (frequency and severity), (2) activities that cause or are limited by breathlessness, (3) impacts on social functioning or psychological disturbances, and (4) total. Lower scores indicated better health.
- Functional data were forced expiratory volume in the first second (FEV_1_), forced vital capacity (FVC), both expressed as absolute values in liters (L) and percentage values (%), FEV_1_/FVC ratio, capacity of alveolo-capillary diffusion of carbon monoxide (DLCO) using the single breath test, both expressed as absolute values in mL∙min^−1^∙mmHg^−1^ and percentage values (%), according to the ATS/ERS guidelines [25].

GraphPad Prism 6.0 was used for statistical analysis. Continuous variables were expressed as mean ± standard deviation (SD) for normally distributed data, or as median with interquartile range (IQR). Normality was assessed using the Shapiro–Wilk test. Comparisons between T1 and T2 were performed using a paired *t*-test for normally distributed data or the non-parametric Wilcoxon signed-rank test for non-normally distributed data. Two-tailed analysis was performed on all data and a *p*-value < 0.05 was considered statistically significant. 

A chi-square test was employed to assess differences in the proportion of patients with more than two exacerbations or at least one exacerbation requiring hospitalization before and after the replacement therapy, as well as the proportion of patients without exacerbations. No formal sample-size calculation was performed owing to the exploratory design and rarity of eligible cases; the cohort size reflects consecutive patients meeting criteria over the study period. Effect sizes with 95% confidence intervals (CIs) were reported for all outcomes. Paired mean differences (95% CI) were computed for SGRQ and lung function (t-based when appropriate); for paired non-normal data (exacerbations), the Hodges–Lehmann median difference with 95% CI was used.

## 3. Results

Among the 34 patients initially selected, 27 were enrolled. Seven patients were excluded from the study due to incomplete SGRQ assessment or respiratory functional data.

The genotypes of patients were the following: 8 PI*MZ, 1 PI*MS, and 18 PI*MR genotypes (4 PI*M/M_wurzburg_, 4 PI*M/P_lowell_, 2 PI*M/M_Procida,_ 2 PI*M/M_whitestable_, 1 PI*M/P_savona_, 1 PI*M/Q0_Perugia_, 1 PI*M/Q0_Cairo_, 1 PI*M/Q0_Ourem_, 1 PI*M/S_munich_, 1 PI*M/I). The median AAT serum level was 88 mg/dl (74–102), indicative of an intermediate deficiency.

All patients were affected by COPD and among them one had a history of recurrent pneumothorax, one had concomitant bronchial asthma, one bronchiectasis, and one a C-ANCA vasculitis.

**Figure 1:**
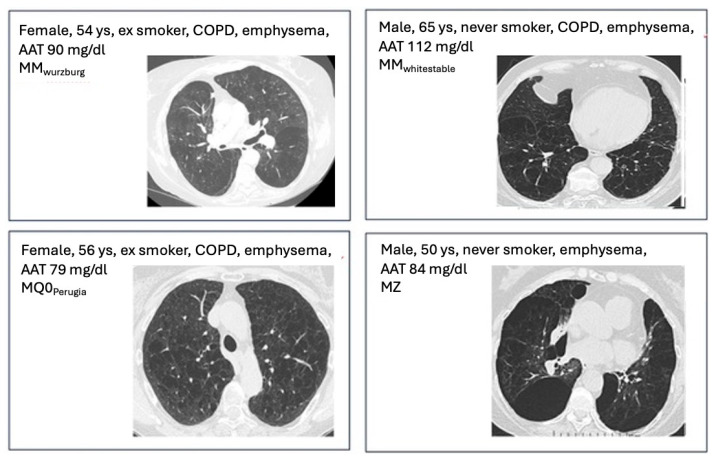
HRCT features and relevant clinical/functional data of some patients with an AAT intermediate deficiency and heterozygous genotype.

The baseline characteristics are shown in Table 1. HRCT features and relevant clinical/functional data of some patients are shown in Figure 1.

### 3.1. Effect of AAT Replacement Therapy on Exacerbations

AT T1 the median (IQR) number of exacerbations in the previous year was 2 (1.5–3); that was significantly reduced by AAT therapy at T2 to 1 (0–1) (*p* < 0.0001).

**Figure 2:**
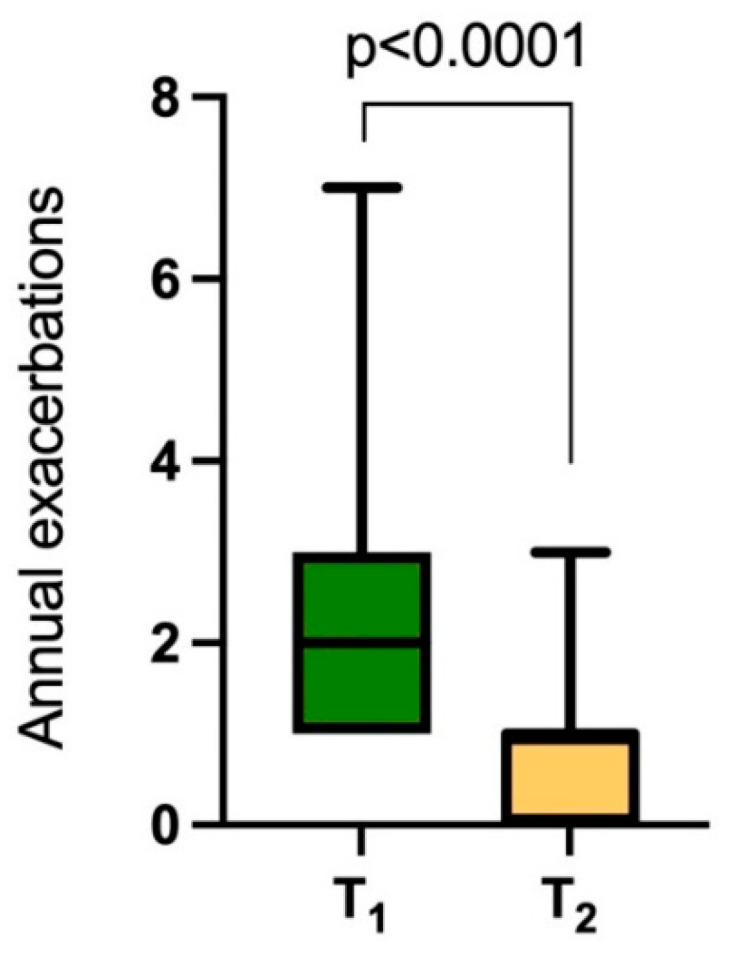
Effectiveness of AAT replacement therapy on total number of exacerbations.

**Figure 3:**
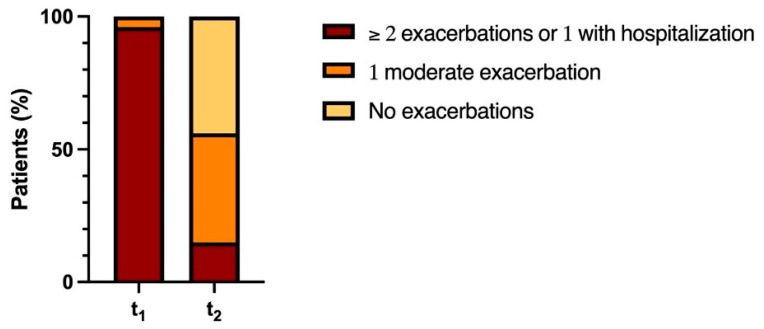
Effect of AAT replacement/augmentation therapy on the percentage of patients with exacerbations.

The percentage of patients with more than two exacerbations or one exacerbation requiring hospitalization decreased from 96% (26 patients) to 15% (3 patients), while we observed an increase in the number of patients with one moderate exacerbation from 4% (1 patient) to 44% (12 patients) and with no exacerbations from 0% to 44% (12 patients). The results are shown in Figure 2 and Figure 3.

### 3.2. Effect of AAT Replacement Therapy on Quality of Life (SGRQ)

Replacement therapy led to a significant improvement in quality of life, with a reduction in the SGRQ total score between T1 and T2 (58.89 ± 16.83 vs. 48.34 ± 21.20, *p* = 0.0039).

**Figure 4:**
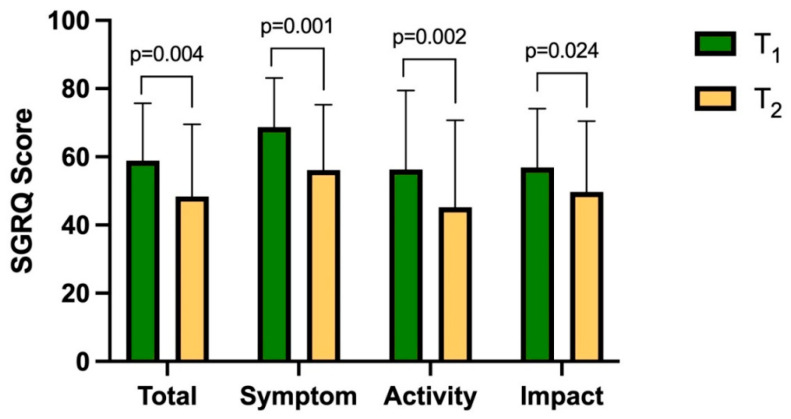
Effectiveness of AAT replacement therapy on the SGRQ domains.

The improvement occurred in each SGRQ domain: symptom (68.68 ± 14.43 vs. 56.15 ± 19.14, *p* = 0.0012), activity (56.29 ± 23.14 vs. 45.22 ± 25.48, *p* = 0.0019), and social impact (56.89 ± 17.27 vs. 49.74 ± 20.75, *p* = 0.024) scores. The results of the SGRQ are shown in Figure 4. Notably, the mean change exceeded the 4-point MCID for COPD, supporting the clinical relevance of the observed improvement.

### 3.3. Effect of AAT Replacement Therapy on Respiratory Function

Replacement therapy did not determine a significant change in FEV_1_ between T1 and T2, neither in the mean absolute values (L) (1.97 ±0.97 vs. 2.04 ±1.05, *p* = 0.27) nor in the mean percentage predicted values (69.61 ± 29.38 vs. 71.73 ± 33.24, *p* = 0.098). Similarly, there was not a significant difference between T1 and T2 in the mean FEV_1_/FVC ratio (64.85 ± 18.73 vs. 62.99 ± 23.44, *p* = 0.49). In the same way, replacement therapy did not significantly change DLCO absolute (5.12 ± 2.17 vs. 5.40 ± 2.85, *p* = 0.95) or percentage predicted values (60.21 ± 21.12 vs. 59.02 ± 22.13, *p* = 0.84).

## 4. Discussion

In this multicenter retrospective cohort of patients with intermediate AATD and COPD/emphysema, 12 months of augmentation therapy was associated with a reduction in annual exacerbations and with a clinically meaningful improvement in health status (SGRQ), while spirometric and DLCO measures did not change significantly.

These findings suggest potential benefits of augmentation therapy in carefully selected intermediate AATD phenotypes; they are hypothesis-generating and do not imply a recommendation for routine treatment beyond current guidelines. One year of replacement therapy was shown to be effective in reducing the rate of exacerbations in a cohort of patients with intermediate AATD with a pathological respiratory phenotype.

Beyond statistical significance, the reduction in exacerbation frequency may be clinically meaningful in this selected intermediate AATD population; this observation, however, should be interpreted cautiously given the retrospective design and lack of a control group. In particular, the shift from a median of two exacerbations (IQR 1.5–3) to one (0–1), along with the increased proportion of patients without exacerbations, is consistent with a potential reduction in exacerbation burden, although causality cannot be inferred.

Regarding this field, although current guidelines do not recommend augmentation therapy in this category of patients, clinicians from different Italian centers decided to prescribe it because of their severe clinical phenotype and recurrence of disease exacerbations. Some authors previously reported that patients with an intermediate deficiency affected by chronic respiratory disease may experience frequent pulmonary exacerbations [26,27,28,29,30,31]. Deviations from guideline recommendations in our cohort reflected case-by-case decisions in patients with disproportionate clinical severity (frequent/severe exacerbations and HRCT emphysema) despite optimized care. The present data should be interpreted as a hypothesis for future trials rather than as evidence to modify guideline indications.

Considering the negative effects of exacerbations on both respiratory functionality and survival rate and the positive safety and efficacy profile of the augmentation therapy in patients with severe AATD, medical doctors decided to prescribe replacement therapy. Although all patients described in the study showed serum AAT levels higher than the putative protective threshold of AAT established at 50 mg/dL, we can argue that a state of neutrophilic hyper-activity can persist in these patients or that the effects of some rare deficient allelic variants on AAT function are not yet defined. Indeed, a previous study demonstrated that more than 85% of AATD patients still experience COPD exacerbations, despite the protective serum AAT levels being reached by the augmentation therapy, suggesting the need for a more aggressive care for the most symptomatic subjects [32]. Although COPD exacerbations are associated with a faster FEV_1_ decline and worse quality of life for AATD patients, a limited number of studies have investigated the effect of AAT therapy on this clinical outcome. In an internet-questionnaire-based survey, Lieberman showed that PI*ZZ patients, receiving augmentation therapy, self-reported a reduced number of annual exacerbations in comparison with those without therapy; as a matter of fact, the majority of patients reported 3−5 annual infections before the beginning of augmentation therapy that decreased to 0−1 during the year of treatment [33]. Barros-Tizón et al. in a multicenter, retrospective observational study involving 127 patients affected by COPD with a severe AAT deficiency demonstrated the efficacy of the augmentation therapy, administered for at least 18 months, in reducing both the incidence and severity of exacerbations, with a consequently significantly lower hospitalization cost [34]. A post hoc analysis of the EXACTLE study demonstrated an effect of the augmentation therapy on the severity of exacerbations but not in its incidence: the study showed a significant reduction in the number of exacerbations requiring corticosteroids or hospitalization, although the overall frequency of exacerbations was unchanged [35]. In a population study, Dahl et al. selected 9187 adults from the Danish general population and followed them for over 21 years. They found that 4.9% patients carried the MZ genotype and that these patients, when compared to MM subjects, had a 50% higher incidence of COPD, as well as a 50% higher chance of exacerbations, hospitalizations, and death from COPD [36].

Exacerbations have a profound impact on patients’ quality of life [37,38,39]. In fact, dyspnea, fatigue, and chronic cough can reduce the ability to carry out daily activities, limiting autonomy and social interactions, affecting not only the physical health but also the emotional wellbeing of patients [40,41,42,43].

COPD exacerbations are a prognostically unfavorable event in the natural history of the disease, causing the worsening of respiratory function and increasing mortality [44,45,46,47,48].

AATD registries showed a relationship between worse SGRQ scores and an elevated exacerbation rate [49,50]. Bernhard et al. in a large cohort of AAT patients with a PI*ZZ genotype showed a significant correlation between SGRQ score and exacerbation frequency in the last two years, with individuals with occupational dust exposure showing a significant worse quality of life [51]. In contrast, Campos et al., in a prospective study, did not observe a significant relationship between SGRQ and exacerbations in one year, and Needham and Stockley observed a significant improvement only in the SGRQ symptoms score in individuals with no or infrequent exacerbations [32,52]. In our small group of AATD patients with an intermediate deficiency, with a heterozygous genotype, all patients self-reported an improvement in their symptoms, activity, and social/emotional impact, and in the overall score of the SGRQ. A recent study showed a significant reduced annual decline in the SGRQ in COPD patients receiving the augmentation therapy in comparison with those on only standard care, although, the authors concluded that their study was not able to reliably confirm if augmentation therapy improved QoL [53]. In our cohort, the mean SGRQ change exceeded the 4-point MCID for COPD, suggesting clinical relevance despite the small sample. This apparent discordance with mixed literature reports [32,52,53] may relate to phenotype enrichment for frequent exacerbators, regression-to-the-mean effects, and the self-reported nature of the SGRQ; hence, the signal requires confirmation in controlled studies.

At present, although replacement therapy is not recommended for patients with an intermediate AATD with a single pathogenic allele or carrying a rare genotype, international guidelines suggest paying attention to these patients. For example, in the latest Italian consensus document, the authors suggested a biennial follow-up for simple, non-pathological AATD heterozygotes, while for heterozygous AATD patients with COPD or liver disease, they suggested a yearly follow-up [54]. However, several case reports have been published regarding successful replacement therapy in patients with an intermediate deficiency carrying one deficient SERPINA-1 allelic variant [26,27,28,55].

Treatment response in intermediate AATD is likely modulated by heterogeneity at both the genotype and phenotype levels. Rare or dysfunctional variants may impair AAT function despite ‘intermediate’ serum concentrations, while clinical modifiers—exacerbation burden, HRCT emphysema pattern/airway involvement, bronchiectasis/asthma overlap, and smoking history—can amplify neutrophil-driven inflammation beyond antiprotease capacity. These factors provide a biologic rationale for phenotype-informed selection and may explain variability in observed benefits.

This study also calls into question the current binary classification of AATD severity, suggesting that intermediate genotypes may warrant therapeutic intervention under specific clinical conditions. Operationally, intermediate AATD candidates for future trials could be classified by (i) exacerbation burden (e.g., ≥2 moderate or ≥1 severe event in the prior year) given its prognostic weight [44,45,46,47,48,49,50,51]; (ii) HRCT evidence of emphysema distribution/airway involvement in capturing structural disease; and (iii) exploratory biomarkers of neutrophil-driven inflammation and AAT functional adequacy to refine risk stratification. This phenotype-based approach may better identify those most likely to benefit than serum AAT levels alone.

The use of AAT replacement/augmentation therapy in clinical practice is frequently in disagreement with current guidelines regarding patients with intermediate defects and varies greatly among AATD specialists with no concordance among them as highlighted in a recent survey, where a significant number of patients with genotype Pi*SZ and almost 10% Pi*MZ were recommended to initiate AT despite a lack of evidence of efficacy in these genotypes. In addition, experts agree that clinical evolution of lung diseases in AATD is difficult to predict on an individual basis and suggest a personalized approach according to the characteristics of the patient [56]. Yet, several studies that we reported underlined that phenotypic expression in patients with intermediate AAT deficiency may vary, and our data are in agreement with the evidence. Furthermore, in our cohort that needed early treatment for patients with intermediate AAT deficiency, we observed a good clinical impact not only on acute medical illness but also on reduction of exacerbations and quality of life.

Furthermore, in our study we did not observe a significant change in the respiratory functional parameters following the AAT therapy. In accordance with our observation, Fraughen et al. in a real-world study documented a survival advantage from intravenous AAT replacement therapy that did not correlate with FEV_1_ decline [20]. This evidence may suggest that in some patients, FEV_1_ monitoring alone may not be sufficient to identify the evolution or the stability of lung damage, probably due to the considerable clinical heterogeneity and the numerous gene variants currently known. Taken together with unchanged spirometry/DLCO, the reduction in exacerbations and improvement in SGRQ support prioritizes exacerbation outcomes and patient-reported health statuses as disease-modifying targets in intermediate AATD, consistent with observational signals that survival and health status may decouple from FEV1 trajectories in selected patients [20,49,50,51].

Our study has numerous limitations related, above all, to the small sample size of patients recruited and their heterogeneity in both genotypes and clinical phenotypes. Moreover, most patients (66.7%) carried rare AAT allelic variants, with different grades in the polymerization of AAT protein at the lung level. This limit is due to the rare prevalence of the pathology and the rare genotype of the patients enrolled for whom augmentation therapy is currently not recommended. Additional limitations include self-reported SGRQs with potential reporting bias, the retrospective design without a control group, a short follow-up (~12 months), and possible between-center variability in ancillary management. Residual confounding (e.g., rehabilitation, adherence, and inhaled therapy optimization) cannot be excluded.

Future research should include multicenter, prospective, controlled trials in intermediate AATD frequent-exacerbator phenotypes, comparing augmentation therapy versus optimized standard care. The primary endpoint should be the annualized rate of moderate/severe exacerbations; key secondary endpoints should include SGRQ (with the 4-point MCID as the threshold for clinical relevance), healthcare utilization, and CT-based emphysema metrics. Stratification by genotype (including rare variants) and smoking history, prespecified biomarker sub-studies, and ≥24 months of follow-up are recommended to ensure adequate power for clinically meaningful effects. We acknowledge that most cited evidence derives from cohorts with severe AATD; our data in intermediate deficiency are exploratory and intended to generate a testable hypothesis for future prospective evaluation in this specific subgroup.

## 5. Conclusions

In this multicenter, retrospective, real-world cohort of patients with intermediate AATD and COPD/emphysema, augmentation therapy was associated with fewer moderate/severe exacerbations and improvement in SGRQ, suggesting a potential benefit in carefully selected phenotypes. Limitations include the retrospective design without a control group, small sample size, short follow-up, heterogeneity across genotypes and centers, and the self-reported nature of the SGRQ. Given these constraints, our findings are hypothesis-generating and should be interpreted with caution. Prospective, adequately powered, controlled trials are needed to confirm these observations and to define phenotype- and biomarker-informed criteria for treatment selection.

## Figures and Tables

**Table 1:** Baseline characteristics of the study population.

Age, mean (SD)	56.70 (13.14)
Sex F, n (%)	12 (44)
BMI, mean (SD)	22.3 (6.9)
Ex smokers, n (%)	20 (75)
No smokers, n (%)	7 (25)
*Clinical phenotype/HRCT Pattern, n (%)*	
COPD/Panlobular and centrilobular emphysema	14 (52)
COPD-Bronchiectasies/Bronchiectasies	6 (22)
*COPD-cANCA vasculitis/Panlobular and centrilobular emphysema*	1 (4)
CPFE/Centrilobular emphysema and fibrosis	1 (4)
COPD-Pneumothorax/Panlobular and centrilobular emphysema	4 (15)
*Comorbidities, n (%)* *Allergic rhinitis* *Chronic rhinosinusitis* *Facioscapulohumeral dystrophy* *Obesity* *Atypical mycobacteriosis* *Ulcerative colitis* *Arterial hypertension*	15 (55) 5 (19) 1 (4) 1 (4) 2 (7) 1 (4) 1 (4) 5 (19)
*Bronchodilator therapy, n (%)* LABA/LAMA/ICS	27 (100)
AAT dosage mg/dL, median (IQR)	88 (74–102)
FEV_1_ % mean (SD)	61.9 (31.3)
FEV_1_/FVC, mean (SD)	64.3 (17.8)
DLCO, %, mean (SD)	62.61 (18.10)

## Data Availability

The research data associated with this paper are available locally at the site, and accessible under approval.

## References

[1] Mulkareddy V., Roman J. (2024). Pulmonary manifestations of alpha 1 antitrypsin deficiency. Am. J. Med Sci..

[2] Miravitlles M., Dirksen A., Ferrarotti I., Koblizek V., Lange P., Mahadeva R., McElvaney N.G., Parr D., Piitulainen E., Roche N. (2017). European Respiratory Society statement: Diagnosis and treatment of pulmonary disease in α1-antitrypsin deficiency. Eur. Respir. J..

[3] Seixas S., Marques P.I. (2021). Known Mutations at the Cause of Alpha-1 Antitrypsin Deficiency an Updated Overview of *SERPINA1* Variation Spectrum. Appl. Clin. Genet..

[4] Sandford A.J. (2020). Alpha-1 Antitrypsin Mutations: Is One Too Many?. Am. J. Respir. Crit. Care Med..

[5] Hazari Y.M., Bashir A., Habib M., Bashir S., Habib H., Qasim M.A., Shah N.N., Haq E., Teckman J., Fazili K.M. (2017). Alpha-1-antitrypsin deficiency: Genetic variations, clinical manifestations and therapeutic interventions. Mutat. Res. Rev. Mutat. Res..

[6] Santangelo S., Scarlata S., Poeta M., Bialas A., Paone G., Incalzi R. (2017). Alpha-1 Antitrypsin Deficiency: Current Perspective from Genetics to Diagnosis and Therapeutic Approaches. Curr. Med. Chem..

[7] de Serres F., Blanco I. (2014). Role of alpha-1 antitrypsin in human health and disease. J. Intern. Med..

[8] Ottaviani S., Gorrini M., Scabini R., Kadija Z., Paracchini E., Mariani F., Ferrarotti I., Luisetti M. (2011). C reactive protein and alpha1-antitrypsin: Relationship between levels and gene variants. Transl. Res..

[9] Ferrarotti I., Piloni D., Filosa A., Ottaviani S., Barzon V., Balderacchi A.M., Corda L., Seebacher C., Magni S., Mariani F. (2025). Clinical features in patients with severe Alpha-1 antitrypsin deficiency due to rare genotypes. Pulmonology.

[10] Aiello M., Fantin A., Longo C., Ferrarotti I., Bertorelli G., Chetta A. (2020). Clinical manifestations in patients with PI*MM_Malton_ genotypes. A matter still unsolved in alpha-1 antitrypsin deficiency. Respirol. Case Rep..

[11] Lieberman J., Colp C. (1990). A role for intermediate, heterozygous alpha 1-antitrypsin deficiency in obstructive lung disease. Chest.

[12] Al Ashry H.S., Strange C. (2017). COPD in individuals with the PiMZ alpha-1 antitrypsin genotype. Eur. Respir. Rev..

[13] Molloy K., Hersh C.P., Morris V.B., Carroll T.P., O’cOnnor C.A., Lasky-Su J.A., Greene C.M., O’nEill S.J., Silverman E.K., McElvaney N.G. (2014). Clarification of the risk of chronic obstructive pulmonary disease in α1-antitrypsin deficiency PiMZ heterozygotes. Am. J. Respir. Crit. Care Med..

[14] Foreman M.G., Wilson C., DeMeo D.L., Hersh C.P., Beaty T.H., Cho M.H., Ziniti J., Curran-Everett D., Criner G., Hokanson J.E. (2017). Genetic Epidemiology of COPD (COPDGene) Investigators. Alpha-1 Antitrypsin PiMZ Genotype Is Associated with Chronic Obstructive Pulmonary Disease in Two Racial Groups. Ann. Am. Thorac. Soc..

[15] Ghosh A.J., Hobbs B.D., Moll M., Saferali A., Boueiz A., Yun J.H., Sciurba F., Barwick L., Limper A.H., Flaherty K. (2022). COPDGeneInvestigators. Alpha-1 Antitrypsin MZ Heterozygosity Is an Endotype of Chronic Obstructive Pulmonary Disease. Am. J. Respir. Crit. Care Med..

[16] Annunziata A., Fiorentino G., Balestrino M., Rega R., Spinelli S., Atripaldi L., Sola A., Massaro F., Calabrese C. (2024). Alpha-1 Antitrypsin PI M Heterozygotes with Rare Variants: Do They Need a Clinical and Functional Follow-Up?. J. Clin. Med..

[17] Fähndrich S., Bernhard N., Lepper P.M., Vogelmeier C., Seibert M., Wagenpfeil S., Bals R. (2017). Exacerbations and duration of smoking abstinence are associated with the annual loss of FEV1 in individuals with PiZZ alpha-1-antitrypsin deficiency. Respir. Med..

[18] Chapman K.R., Burdon J.G.W., Piitulainen E., Sandhaus R.A., Seersholm N., Stocks J.M., Stoel B.C., Huang L., Yao Z., Edelman J.M. (2015). Intravenous augmentation treatment and lung density in severe α1 antitrypsin deficiency (RAPID): A randomised, double-blind, placebo-controlled trial. Lancet.

[19] McElvaney N.G., Burdon J., Holmes M., Glanville A., Wark P.A.B., Thompson P.J., Hernandez P., Chlumsky J., Teschler H., Ficker J.H. (2017). Long-term efficacy and safety of α1 proteinase inhibitor treatment for emphysema caused by severe α1 antitrypsin deficiency: An open-label extension trial (RAPID-OLE). Lancet Respir. Med..

[20] Fraughen D.D., Ghosh A.J., Hobbs B.D., Funk G.-C., Meischl T., Clarenbach C.F., Sievi N.A., Schmid-Scherzer K., McElvaney O.J., Murphy M.P. (2023). Augmentation therapy for severe Alpha-1 antitrypsin deficiency improves survival and is decoupled from Spirometric decline-a multinational registry analysis. Am. J. Respir. Crit. Care Med..

[21] Bernhard N., Lepper P.M., Vogelmeier C., Seibert M., Wagenpfeil S., Bals R., Fähndrich S. (2017). Intensive smoking diminishes the differences in quality of life and exacerbation frequency between the alpha-1-antitrypsin deficiency genotypes PiZZ and PiSZ. Respir. Med..

[22] Miravitlles M., Anzueto A., Barrecheguren M. (2023). Nine controversial questions about augmentation therapy for alpha-1 antitrypsin deficiency: A viewpoint. Eur. Respir. Rev..

[23] Balderacchi A.M., Barzon V., Ottaviani S., Corino A., Zorzetto M., Wencker M., Corsico A.G., Ferrarotti I. (2021). Comparison of different algorithms in laboratory diagnosis of alpha1-antitrypsin deficiency. Clin. Chem. Lab. Med..

[24] Anthonisen N.R., Manfreda J., Warren C.P.W., Hershfield E.S., Harding G.K.M., Nelson N.A. (1987). Antibiotic therapy in exacerbations of chronic obstructive pulmonary disease. Ann. Intern. Med..

[25] Bhakta N.R., McGowan A., Ramsey K.A., Borg B., Kivastik J., Knight S.L., Sylvester K., Burgos F., Swenson E.R., McCarthy K. (2023). European Respiratory Society/American Thoracic Society technical statement: Standardisation of the measurement of lung volumes, 2023 update. Eur. Respir. J..

[26] Blanco I., Canto H., Flóres J., Camblor C., Cárcaba V., De Serres F., Janciauskiene S., Bustillo E. (2008). Long-term augmentation therapy with alpha-1 antitrypsin in an MZ-AAT severe persistent asthma. Monaldi. Arch. Chest. Dis..

[27] Annunziata A., Ferrarotti I., Lanza M., Cauteruccio R., Di Spirito V., Fiorentino G. (2020). Alpha 1 antitrypsin deficiency and intermediate risk: Case of a heterozygote for the MWurzburg allele. Rass. Patol. Dell’apparato Respir..

[28] Annunziata A., Coppola A., Coni P., Fiorentino G. (2021). Intermediate Alpha-1 Antitrypsin Deficiency Can Play a Role in Pulmonary Exacerbation?. Glob. J. Respir. Care.

[29] Barjaktarevic I., Miravitlles M. (2021). Alpha-1 antitrypsin (AAT) augmentation therapy in individuals with the PI*MZ genotype: A pro/con debate on a working hypothesis. BMC Pulm. Med..

[30] Hernández-Pérez J.M., Martín-González E., González-Carracedo M.A. (2023). Alpha-1 Antitrypsin Deficiency and SERPINA1 Variants Could Play a Role in Asthma Exacerbations. Arch. Bronc..

[31] Martín-González E., Hernández-Pérez J.M., Pérez J.A.P., Pérez-García J., Herrera-Luis E., González-Pérez R., González-González O., Mederos-Luis E., Sánchez-Machín I., Poza-Guedes P. (2025). Alpha-1 antitrypsin deficiency and Pi*S and Pi*Z SERPINA1 variants are associated with asthma exacerbations. Pulmonology.

[32] Campos M.A., Alazemi S., Zhang G., Wanner A., Salathe M., Baier H., Sandhaus R.A. (2009). Exacerbations in subjects with alpha-1 antitrypsin deficiency receiving augmentation therapy. Respir. Med..

[33] Lieberman J. (2000). Augmentation therapy reduces frequency of lung infections in antitrypsin deficiency: A new hypothesis with supporting data. Chest.

[34] Barros-Tizón J.C., Torres M.L., Blanco I., Martínez M.T. (2012). Investigators of the rEXA study group. Reduction of severe exacerbations and hospitalization-derived costs in alpha-1-antitrypsin-deficient patients treated with alpha-1-antitrypsin augmentation therapy. Ther. Adv. Respir. Dis..

[35] Dirksen A., Piitulainen E., Parr D.G., Deng C., Wencker M., Shaker S.B., Stockley R.A. (2009). Exploring the role of CT densitometry: A randomised study of augmentation therapy in alpha1-antitrypsin deficiency. Eur. Respir. J..

[36] Dahl M., Tybjærg-Hansen A., Lange P., Vestbo J., Nordestgaard B.G. (2002). Change in lung function and morbidity from chronic obstructive pulmonary disease in alpha1-antitrypsin MZ heterozygotes: A longitudinal study of the general population. Ann. Intern. Med..

[37] Smith D.J., Ellis P.R., Turner A.M. (2021). Exacerbations of Lung Disease in Alpha-1 Antitrypsin Deficiency. Chronic Obstr. Pulm. Dis..

[38] Choate R., Sandhaus R.A., Holm K.E., Mannino D.M., Strange C. (2022). Patient-Reported Pulmonary Symptoms, Exacerbations, and Management in a Cohort of Patients with Alpha-1 Antitrypsin Deficiency. Chronic Obstr. Pulm. Dis..

[39] Hurst J.R., Skolnik N., Hansen G.J., Anzueto A., Donaldson G.C., Dransfield M.T., Varghese P. (2020). Understanding the impact of chronic obstructive pulmonary disease exacerbations on patient health and quality of life. Eur. J. Intern. Med..

[40] Guo J., Chen Y., Zhang W., Tong S., Dong J. (2020). Moderate and severe exacerbations have a significant impact on health-related quality of life, utility, and lung function in patients with chronic obstructive pulmonary disease: A meta-analysis. Int. J. Surg..

[41] MacLeod M., Papi A., Contoli M., Beghé B., Celli B.R., Wedzicha J.A., Fabbri L.M. (2021). Chronic obstructive pulmonary disease exacerbation fundamentals: Diagnosis, treatment, prevention and disease impact. Respirology.

[42] Quadflieg K., Machado A., de Lima F.F., Dederen A., Daenen M., Ruttens D., Thomeer M., Spruit M.A., Burtin C. (2023). Physical status, symptoms and health-related quality of life during a severe exacerbation of COPD: Recovery and discriminative capacity for future events. Respir. Med..

[43] Anandan J., Dwivedi D.P., Govindaraj V. (2023). Clinical phenotypes of COPD and their impact on quality of life: A cross-sectional study. Respir. Med..

[44] Suissa S., Dell’ANiello S., Ernst P. (2012). Long-term natural history of chronic obstructive pulmonary disease: Severe exacerbations and mortality. Thorax.

[45] Halpin D.M., Decramer M., Celli B., Kesten S., Liu D., Tashkin D.P. (2012). Exacerbation frequency and course of COPD. Int. J. Chronic Obstr. Pulm. Dis..

[46] Williams N.P., A Coombs N., Johnson M.J., Josephs L.K., A Rigge L., Staples K.J., Thomas M., Wilkinson T.M. (2017). Seasonality, risk factors and burden of community-acquired pneumonia in COPD patients: A population database study using linked health care records. Int. J. Chronic Obstr. Pulm. Dis..

[47] Whittaker H., Rubino A., Müllerová H., Morris T., Varghese P., Xu Y., De Nigris E., Quint J.K. (2022). Frequency and severity of exacerbations of COPD associated with future risk of exacerbations and mortality: A UK routine health care data study. Int. J. Chronic Obstr. Pulm. Dis..

[48] Negro R.W.D. (2019). COPD: The annual cost-of-illness during the last two decades in Italy, and its mortality predictivity power. Healthcare.

[49] Stockley R.A. (2016). Alpha-1 Antitrypsin Deficiency: Phenotypes and Quality of Life. Ann. Am. Thorac. Soc..

[50] Gauvain C., Mornex J.-F., Pison C., Cuvelier A., Balduyck M., Pujazon M.-C., Fournier M., AitIlalne B., Thabut G. (2015). Health-related quality of life in patients with alpha-1 antitrypsin deficiency: The French experience. COPD.

[51] Bernhard N., Lepper P.M., Vogelmeier C., Seibert M., Wagenpfeil S., Bals R., Fähndrich S. (2017). Deterioration of quality of life is associated with the exacerbation frequency in individuals with alpha-1-antitrypsin deficiency-analysis from the German Registry. Int. J. Chronic Obstr. Pulm. Dis..

[52] Needham M., Stockley R.A. (2005). Exacerbations in α_1_-antitrypsin deficiency. Eur. Respir. J..

[53] Stockley R.A., Edgar R.G., Starkey S., Turner A.M. (2018). Health status decline in α-1 antitrypsin deficiency: A feasible outcome for disease modifying therapies?. Respir. Res..

[54] Aliberti S., Amati F., Annunziata A., Arcoleo F., Baderna P., Bini F., Carraro C.F., Iannacci L., Cicero S.L., Passalacqua G. (2022). Diagnosis and management of patients with α1-antitrypsin deficiency: An Italian perspective. Minerva Respir. Med..

[55] Kueppers F. (2021). Clinical presentations of four patients with rare Alpha 1 Antitrypsin variants identified in a single US center. Respir. Med. Case Rep..

[56] Greulich T., Albert A., Cassel W., Boeselt T., Peychev E., Klemmer A., Ferreira F.P., Clarenbach C., Torres-Duran M.L., Turner A.M. (2022). Opinions and Attitudes of Pulmonologists About Augmentation Therapy in Patients with Alpha-1 Antitrypsin Deficiency. A Survey of the EARCO Group. Int. J. Chronic Obstr. Pulm. Dis..

